# Digital reconstructed radiography with multiple color image overlay for image-guided radiotherapy

**DOI:** 10.1093/jrr/rrv002

**Published:** 2015-02-11

**Authors:** Shinichi Yoshino, Kentaro Miki, Kozo Sakata, Yuko Nakayama, Kouichi Shibayama, Shinichiro Mori

**Affiliations:** 1 Department of Radiation Oncology, Kanagawa Cancer Center, 2-3-2, Yokohama-shi, Kanagawa, Japan; 2 Research Center for Charged Particle Therapy, National Institute of Radiological Sciences, 4-9-1 Anagawa, Chiba, Japan

**Keywords:** charged particle beam, positional accuracy, setup, image processing

## Abstract

Registration of patient anatomical structures to the reference position is a basic part of the patient set-up procedure. Registration of anatomical structures between the site of beam entrance on the patient surface and the distal target position is particularly important. Here, to improve patient positional accuracy during set-up for particle beam treatment, we propose a new visualization methodology using digitally reconstructed radiographs (DRRs), overlaid DRRs, and evaluation of overlaid DRR images in clinical cases. The overlaid method overlays two DRR images in different colors by dividing the CT image into two CT sections at the distal edge of the target along the treatment beam direction. Since our hospital uses fixed beam ports, the treatment beam angles for this study were set at 0 and 90 degrees. The DRR calculation direction was from the X-ray tube to the imaging device, and set to 180/270 degrees and 135/225 degrees, based on the installation of our X-ray imaging system. Original and overlaid DRRs were calculated using CT data for two patients, one with a parotid gland tumor and the other with prostate cancer. The original and overlaid DRR images were compared. Since the overlaid DRR image was completely separated into two regions when the DRR calculation angle was the same as the treatment beam angle, the overlaid DRR visualization technique was able to provide rich information for aiding recognition of the relationship between anatomical structures and the target position. This method will also be useful in patient set-up procedures for fixed irradiation ports.

## INTRODUCTION

Thanks to recent progress in radiotherapy, including improvements in irradiation technique and dose calculation, survival rates in cancer treatment have increased [1]. Intensity-modulated radiotherapy using a photon beam or a particle beam allows delivery of the treatment beam to the tumor while minimizing excessive dose to normal tissues [2, 3]. Because the reproducibility of patient position is not perfect, however, patient positional accuracy, including both intrafractional and interfractional changes, remains a major problem. To cope with these confounders, several hospitals routinely use image-guided radiotherapy in clinical care [4].

Patient set-up is conventionally performed using a laser localizer. Recent improvements in high conformal irradiation treatment require high positioning accuracy. To achieve this, one recent patient positional system uses X-ray images in manual and/or automatic mode [5]. The concept of a beam's eye view provides an indication of the geometrical parameters of a given viewpoint. It is practical and useful in understanding the treatment beam's position and target [6], and is used widely in treatment planning and patient set-up. Some recent installations of patient positional systems have been done in an oblique direction to avoid beam collision with the irradiation system. Although anatomical structures proximal to the target can affect dose distribution, and should be preferentially registered to the reference position, anatomical structure images are more difficult to register in oblique than in horizontal or vertical DRR images. Patient positioning with X-ray images uses digitally reconstructed radiographs (DRRs) as the reference images by projecting CT data from the same angle as the X-ray system installation.

To solve the imperfect reproducibility of patient position, we propose use of the overlaid DRR image. This image includes target shape and position, and its rich information aids recognition of the relationship between patient anatomical structures and target position.

Here, we introduce the overlaid DRR visualization method and describe its clinical advantages in the head and pelvic regions.

## MATERIALS AND METHODS

### Layout of the treatment room

Two treatment rooms in our hospital are equipped with vertical (= 0 degree) and horizontal (= 90 degree) beam ports for carbon-ion scanning treatment [7, 8]. These rooms also include a selective compliance assembly robot arm–type treatment couch to improve patient positional accuracy. A pair of X-ray imaging systems includes an X-ray tube and flat panel detector (FPD) in each of the vertical and horizontal directions for patient positional verification. Respective FPDs are installed at the front of the irradiation port. X-ray images obtained by the X-ray imaging system are not beam's eye view, but two oblique X-ray fluoroscopic units for real-time tumor tracking during treatment are installed on the side of the vertical irradiation port. This system is also available for patient positional verification.

In the third treatment room, a gantry port and X-ray fluoroscopic units are installed on the side of the irradiation port. In the gantry treatment room, X-ray images can be acquired at multiple angles.

### Image acquisition

Two patients, one with a parotid gland tumor and the second with prostate cancer, were randomly selected from our patients undergoing carbon-ion scanning beam treatment. All patients were informed of the contents of the study and gave consent to participate, and the study was approved by the institutional review board of our institution. All patients were fixed by immobilization with a shell device to improve positional reproducibility [9]. CT data were acquired in helical mode with a multi-slice CT (Aquilion LB, Toshiba Medical Systems, Japan) under free-breathing conditions. The reconstructed slice thickness was 2.0 mm with 16 detectors owing to the slice collimation of 2.0 mm.

### DRR computation algorithm

General DRR computation integrates CT voxel values along the X-ray projection ray through the whole CT dataset. To minimize calculation time, we implemented a Siddon's ray tracing algorithm, which calculates the radiological path length by determining the intercepts of the ray [10]. Technical details are reported elsewhere [11].

**Fig. 1.**
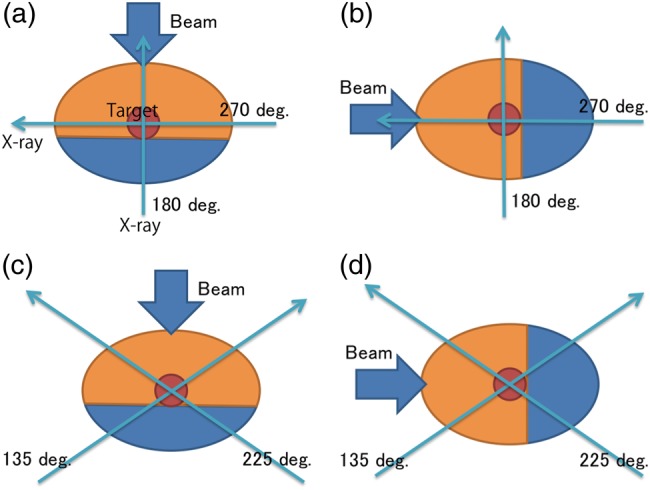
Two DRR images were calculated by dividing CT data at the distal edge of the target along the treatment beam direction and visualized with image overlay. The orange and blue regions are the proximal and distal sides of the target (red circle), respectively. DRR image calculation direction was 180 and 270 degrees for the (**a**) vertical and (**b**) horizontal treatment beam directions, and 135 and 225 degrees for the (**c**) vertical and (**d**) horizontal treatment beam directions, respectively.

Our proposed DRR visualization method (overlaid DRR) is based on the overlay of two DRR images in different colors. CT data are divided into two sections; the first CT section (orange region in Fig. 1) is from the patient surface to the distal edge of the target, and the second section (blue region in Fig. 1) is from the distal edge of the target to the end of the CT data along the treatment beam direction. DRR images are calculated using the respective sections from the appropriate X-ray direction. The two DRR images are then overlaid in different colors; in this study, the proximal and distal sides of the target were colored orange and blue, respectively, to emphasize the anatomical structures located in the proximal side of the target.

For the X-ray system installed in the vertical and horizontal directions, the DRR image calculated from the perpendicular to the treatment beam angle is clearly visualized by separation into two different colors (DRR for 270 degrees in Fig. 1a and DRR for 180 degrees in Fig. 1b). For the X-ray system installed in oblique directions, in contrast, the DRR calculation direction is not perpendicular to the treatment beam direction.

### Image evaluation

Original and overlaid DRR images were calculated from 180/270 degrees and 135/225 degrees, respectively. The treatment beam angle was set to 0 and 90 degrees, which closely reflect clinical situations, and CT data were accordingly divided into two sections along the vertical and horizontal directions (Fig. 1).

Because the purpose of our visualization methodology is to improve patient set-up accuracy and explore clinical benefits, we calculated DRR images for sample head and pelvic cases from a particle therapy standpoint. Quantification analysis was not performed.

## RESULTS AND DISCUSSION

### Head and neck case

**Fig. 2.**
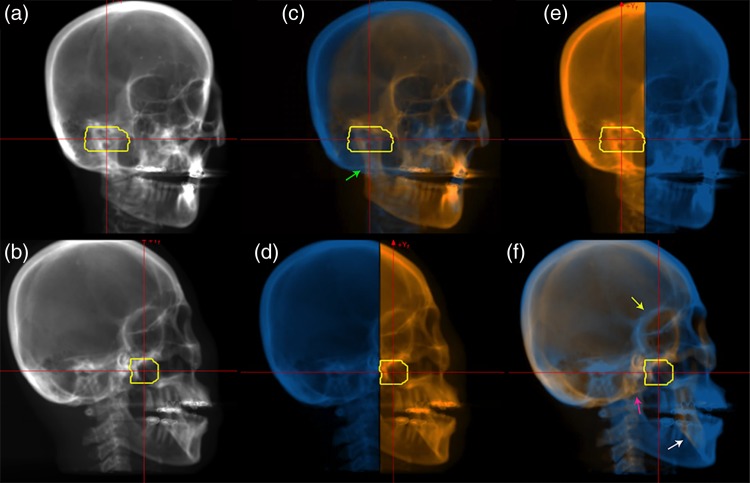
DRR head images calculated from 180 (upper panel) and 270 degrees (lower panel). Original DRR images are shown in (**a**) and (**b**). Overlaid DRR images with a (**c**) (**d**) vertical treatment beam (from right side in Fig. 2d) and (**e**) (**f**) horizontal treatment beam direction (from left side in Fig. 2e). Orange and blue regions show proximal and distal sides of the target, respectively. Yellow lines show the planning target volume. The treatment couch was rotated 10 degrees.

Original and overlaid DRR images for DRR calculation angles of 180 and 270 degrees are shown in Fig. 2. Since the tumor was located close to the left parotid gland and the treatment couch was rotated 10 degrees to extend beam angle selection, bone structures were projected in an oblique direction, even though the DRR calculation angle was not oblique (Fig. 2). In verifying the patient position, medical staff focus on the relationship between patient anatomical sites and the target position. Nevertheless, these are difficult to understand using the original DRR images (Fig. 2a and b). In particular, the target region in the vertical DRR image was overlapped by the mandibular ramus and the occipital bone, and upper and lower jaw positional reproducibility was not high, even though the patient had a bite block device. In this case, patient set-up should not be carried out by focusing on the occipital bone. The overlaid DRR image clearly separated these structures for irradiation with the vertical treatment beam (green arrow in Fig. 2c).

For irradiation with the horizontal treatment beam, the supraorbital and infraorbital rims were colored blue and orange, respectively. However, medical staff easily recognized that these structure positions should not be considered separately (yellow arrow in Fig. 2f). As in Fig. 2c, the mandibular ramus and the area around the mandibular fossa were successfully separated for visualization (red arrow in Fig. 2f).

The overlaid DRR images in Fig. 2d and e were completely separated into two regions when the DRR calculation angle was perpendicular to the treatment beam.

**Fig. 3.**
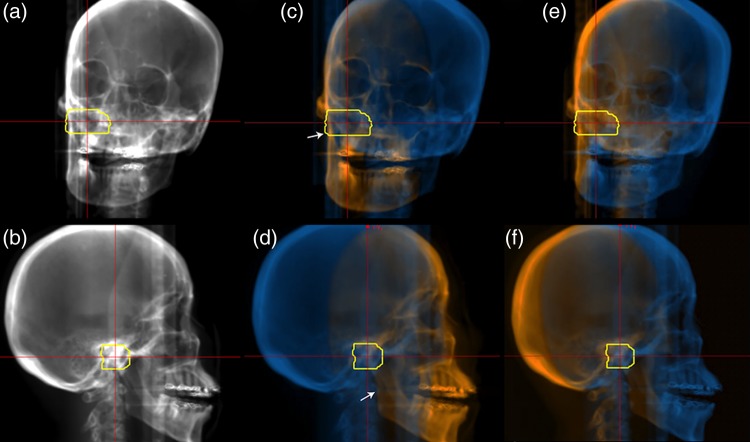
DRR head images calculated from 135 (upper panel) and 225 degrees (lower panel). Original DRR images are shown in (**a**) and (**b**). Overlaid DRR images with a (**c**) (**d**) vertical treatment beam (from right side in Fig. 3d) and (**e**) (**f**) horizontal treatment beam direction (from left side in Fig. 3e). Orange and blue regions show proximal and distal sides of the target, respectively. Yellow lines show the planning target volume. The treatment couch was rotated 10 degrees.

For the DRR calculation angle from oblique directions, the overlaid DRR images were substantially more informative than the original images in recognizing the relationship between anatomical structures and the target position (Fig. 3). The overlaid DRR images clearly separated the lower jaw structure at the proximal and distal side of the target (white arrows in Fig. 3).

### Pelvic case

**Fig. 4.**
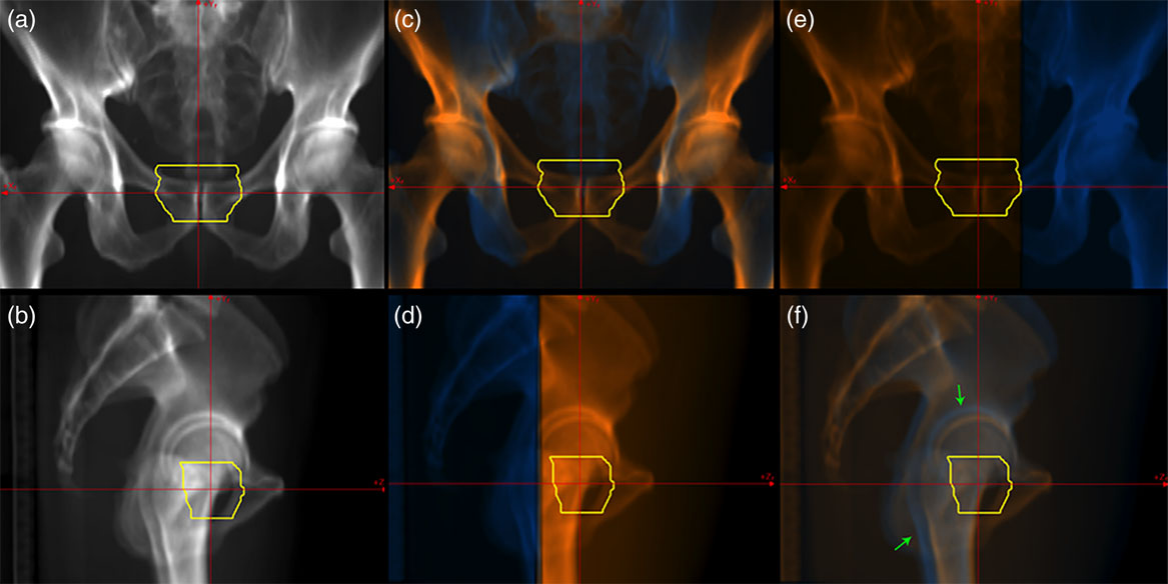
DRR pelvic images calculated from 180 (upper panel) and 270 degrees (lower panel). Original DRR images are shown in (**a**) and (**b**). Overlaid DRR images with a (**c**) (**d**) vertical treatment beam (from right side in Fig. 4d) and (**e**) (**f**) horizontal treatment beam direction (from left side in Fig. 4e). Orange and blue regions show proximal and distal sides of the target, respectively. Yellow lines show the planning target volume.

Anatomical structures in the DRR pelvic image calculated from 180 degrees are relatively easy to recognize (Fig. 4a). For vertical treatment beam irradiation, however, the overlaid DRR images calculated from 180 and 270 degrees were not particularly informative (Fig. 4c and d), especially in the DRR image calculated from 180 degrees. This was because the target was located anterior to the inferior ramus of the pubic bone. Our protocol for prostate treatment uses carbon-ion beam irradiation from the lateral directions, and dose distribution is strongly affected by the position of the femur. However, anatomical structures on the left and right sides of the patient were observed as overlapped in DRR calculated from 270 degrees. The overlaid DRR separated the left and right femur bones (green arrows in Fig. 4f). Medical staff registered the femur bone at the proximal side of the target only. As in the previous case, the overlaid DRR images calculated from the angle perpendicular to the treatment beam direction separated the regions completely (Fig. 4d and e); however, they could be useful for patient set-up.

**Fig. 5.**
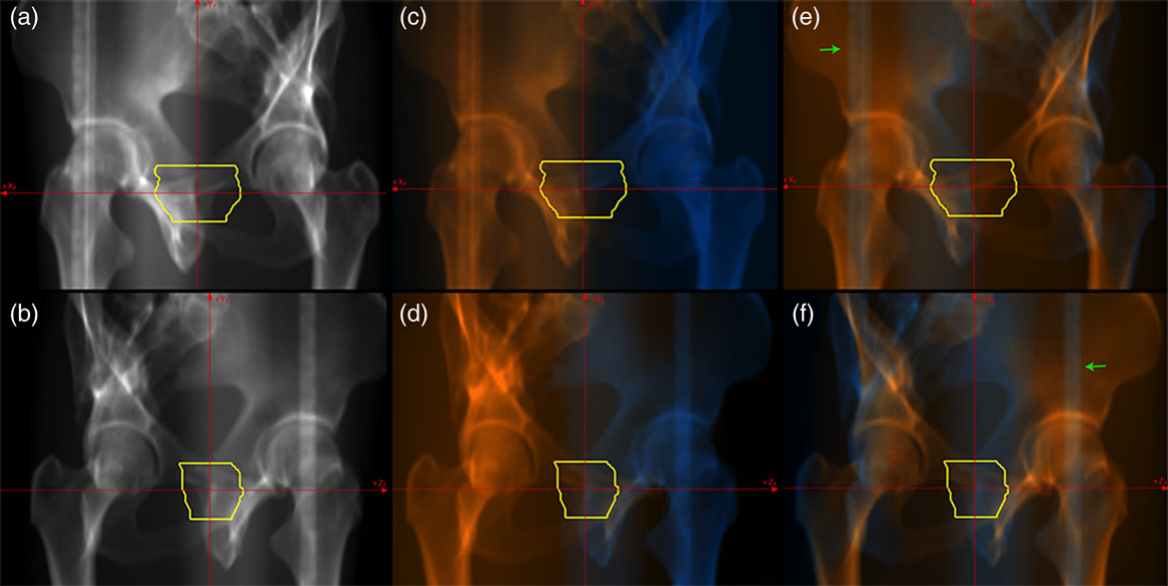
DRR pelvic images calculated from 135 (upper panel) and 225 degrees (lower panel). Original DRR images are shown in (**a**) and (**b**). Overlaid DRR images with a (**c**) (**d**) vertical treatment beam (from right side in Fig. 5d) and (**e**) (**f**) horizontal treatment beam direction (from left side in Fig. 5e). Orange and blue regions show proximal and distal sides of the target, respectively. Yellow lines show the planning target volume.

With regard to the oblique DRR calculation angle, the target was overlapped with the superior ramus of the pubic bone and its surrounding bones in both the left and right sides of the patient (Fig. 5a and b). These pelvic bones are not completely rigid, but have a very limited degree of mobility provided by the sacroiliac joint and pubic symphysis. When these pelvic bones were not completely registered to the reference position due to patient position, pelvic bones at the proximal side of the target were selectively registered using the overlaid DRR images (Fig. 5e–f).

Despite use of custom-made immobilization, patients were not always laid on the treatment couch in the same position in each treatment fraction, and the relationship between patient and treatment couch positional reproducibility is not high. Our treatment couch consists mostly of low-density materials, but the bar frame at both sides of the couch includes high-density material to minimize deflection due to patient weight. These high-density materials were visualized on the DRR images, and their overlap with the patient might affect patient set-up accuracy in certain cases. In the prostate treatment case, DRR images calculated from 180 and 270 degrees did not include these high-density regions, whereas those from oblique angles did. For the vertical treatment beam, the treatment couch and anatomical structures were at the proximal side of the target (Fig. 5c and d).

For the horizontal beam, the treatment couch was located at the distal side of the target and should be removed for patient set-up, and it also overlapped with anatomical structures at the proximal side of the target (Fig. 5a and b). This overlap on the proximal side is somewhat difficult to understand, particularly in the original DRR from an oblique direction. In the overlaid DRR images, however, the treatment couch at the distal side of the target was successfully separated from anatomical structures (green arrows in Fig. 5e and f). This ability to remove the treatment couch should improve patient set-up accuracy for the 2D–3D auto registration function.

## CONCLUSIONS

Our proposed image visualization methodology, the ‘overlaid DRR image’, provides rich information to aid medical staff in recognizing the relationship between anatomical structures and the target position. Although the present paper describes its use in only two patients, this technique should improve patient set-up accuracy and minimize procedure time by registering anatomical sites within the proximal side of the target. As a result, the overlaid DRR image may improve dose conformation to the target, particularly in particle beam therapy. Finally, although this paper introduces the overlaid DRR visualization technique on the basis of our treatment room geometry, implementation with the gantry irradiation port in particle and photon beam therapies should also prove useful.
